# Effects of thermal annealing on localization and strain in core/multishell GaAs/GaNAs/GaAs nanowires

**DOI:** 10.1038/s41598-020-64958-6

**Published:** 2020-05-19

**Authors:** Roman M. Balagula, Mattias Jansson, Mitsuki Yukimune, Jan E. Stehr, Fumitaro Ishikawa, Weimin M. Chen, Irina A. Buyanova

**Affiliations:** 1 Department of Physics, Chemistry and Biology, Linköping University, 581 83 Linköping, Sweden; 2 Graduate School of Science and Engineering, Ehime University, 790-8577 Matsuyama, Japan

**Keywords:** Nanowires, Quantum dots, Semiconductors, Electronic properties and materials

## Abstract

Core/shell nanowire (NW) heterostructures based on III-V semiconductors and related alloys are attractive for optoelectronic and photonic applications owing to the ability to modify their electronic structure via bandgap and strain engineering. Post-growth thermal annealing of such NWs is often involved during device fabrication and can also be used to improve their optical and transport properties. However, effects of such annealing on alloy disorder and strain in core/shell NWs are not fully understood. In this work we investigate these effects in novel core/shell/shell GaAs/GaNAs/GaAs NWs grown by molecular beam epitaxy on (111) Si substrates. By employing polarization-resolved photoluminescence measurements, we show that annealing (i) improves overall alloy uniformity due to suppressed long-range fluctuations in the N composition; (ii) reduces local strain within N clusters acting as quantum dot emitters; and (iii) leads to partial relaxation of the global strain caused by the lattice mismatch between GaNAs and GaAs. Our results, therefore, underline applicability of such treatment for improving optical quality of NWs from highly-mismatched alloys. They also call for caution when using *ex-situ* annealing in strain-engineered NW heterostructures.

## Introduction

III-V semiconductor nanowires (NW) hold great potential for numerous applications in electronics and photonics. The advantages provided by the one-dimensional (1D) geometry of such structures include but are not limited to: a large surface-to-volume ratio beneficial for sensor applications^1–3^; controllable density of states due to electron confinement leading to flexibility in optical and electrical properties^4,5^; strong photon confinement in naturally-formed NW cavities and waveguides^6–8^; relaxed lattice match requirements allowing NW growth on foreign substrates; etc. A simple way of significantly changing band structure by alloying in ternary or quaternary materials is also a merit. Moreover, an important advantage of the NW architecture is the ability to modify their electronic properties via strain engineering^9–11^, owing to the fact that related heterostructures can withstand high elastic strain that is not achievable in epitaxially-grown lattice-mismatched films.

Among III-V NWs, GaAs-based NWs are extensively studied and can now be fabricated in complex architectures, including axial or radial (core/shell) heterostructures. This has led to the development of numerous prototype device structures, such as efficient lasers^12–14^, HEMTs^15,16^, LEDs^17,18^, photodetectors^19,20^ and solar cells^21–24^ from high-quality GaAs, GaAs/AlGaAs and In(Ga)As/GaAs NW heterostructures. It was discovered over the past years that the bandgap of GaAs-based compounds in the 0D, 2D and 3D geometries can be significantly tailored by adding nitrogen, forming the so-called dilute nitrides. For example, introduction of only 2% of nitrogen can reduce the bandgap energy of the GaNAs alloy by 0.3 eV, bringing it closer to the spectral range for telecommunication applications^25^. This bandgap engineering is accompanied by other attractive modifications of the electronic structure including splitting of the conduction band (CB) into two subbands^26,27^, an increase of the electron effective mass^28–32^, unusual defect-mediated spin functionalities^33–36^, etc. Therefore, development of heterostructured NWs based on dilute nitride alloys is promising for a great variety of applications in optoelectronics and photonics^37^, which will combine the advantages offered by the 1D geometry and dilute nitrides. For example, near-infrared lasing from GaNAs-based core/shell NW heterostructures has recently been realized^38,39^, where the lasing wavelength could be extended to 1 μm by using GaNAs alloys with a nitrogen composition [N] as low as 2.5%. A nano-photonic structure of GaNAs nanodisks-in-GaAs nanopillars has been shown^36^ to represent an efficient nano-sized interface for spin-to-photon conversion at room temperature, which can be utilized in future nanoscale spin-photonics and quantum communication networks. Adding N also reduces the lattice constant of the GaNAs alloy opening possibilities for strain engineering in GaAs/GaNAs NW heterostructures. However, due to a large difference in sizes between the N and As atoms and the known tendency of nitrogen to form various clusters, GaNAs can also experience local strain. The local strain may dominate over the global strain in the regions of such short-range fluctuations in the N content and can, therefore, determine their local electronic structure, as was shown previously in GaNAs/GaAs core/shell NWs with [N] = 0.5%^40^.

Fabrication of complex NW heterostructures often involves post-growth annealing, which may represent a necessary step during device fabrication and is used to enhance transport and optical properties of the active material, due to annealing-out of grow-in defects^41–44^ and improved alloy uniformity^45–47^. However, effects of such post-growth annealing on the global and local strain in NW heterostructures are yet to be understood. In this paper we analyze these effects in novel GaAs/GaNAs/GaAs core/shell/shell (CSS) NW heterostructures by utilizing polarization-resolved photoluminescence (PL) spectroscopy.

## Samples and Methods

**Figure 1:**
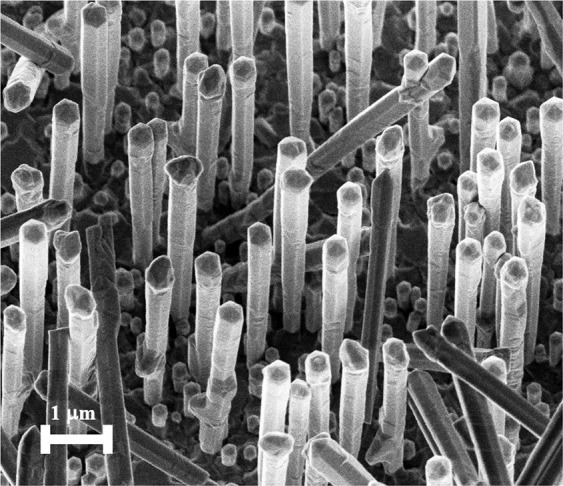
Characteristic SEM image of as-grown GaAs/GaNAs/GaAs CSS NW ensemble.

The investigated GaAs/GaNAs/GaAs core/shell/shell (CSS) NWs were grown by self-catalyzed molecular beam epitaxy (MBE) on Si substrates. Nitrogen composition [N] in the active GaNAs shell was 2%, as determined based on the spectral position of the room-temperature band-to-band emission using the band-anticrossing (BAC) model^27,37^. This N composition represents the maximum reported value for NWs with good optical quality^48^. Details of the growth process can be found in^49^. The NW arrays are found to be dense and uniform, with most of the wires (3–5 μm long) standing vertically or slightly tilted from the vertical axis. This can be seen from Fig. 1, where a representative scanning electron microscopy (SEM) image of the as-grown NW array is shown. Single NWs have a hexagonal cross-section with well-defined facets suggesting that they were epitaxially grown along the [111] crystallographic axis. Total diameters of the NWs range between 270 and 350 nm, with a core diameter of 170–200 nm, and inner and outer shell thicknesses of 50–75 nm each, as deduced from the cross-sectional scanning transmission electron microscopy (STEM) data. The STEM measurements also revealed the formation of an extra GaNAs layer with a small N content of 0.3–0.5%. This thin and optically inactive layer is located close to the edge of the GaAs core and is likely formed due to the active nitrogen penetrating the closed shutter of the plasma^49–51^. After the growth, the sample was split in several pieces and some pieces were annealed for 15 minutes in the growth chamber under As_2_ overpressure at 680 °C.

Optical characterization was performed by means of micro-PL (μPL) measurements on NW arrays and single NWs mechanically transferred onto a Si substrate. Samples were mounted in a flow cryostat cooled down with liquid helium. The sample was excited with a 660 nm solid-state laser in a backscattering geometry via a microscope objective with 50x magnification and 0.5 numerical aperture. The same objective was used to collect the emitted light, which was spectrally analyzed by a grating monochromator and detected by a LN_2_-cooled InGaAs linear array detector. The excitation spot was roughly 1 μm in diameter. Linear polarization measurements were performed in the same experimental scheme by adding a rotatable half-wave plate followed by a fixed linear polarizer before an entrance slit of the monochromator. The polarization degree P was calculated as *P* = 100% ∙ (*I*_⊥_ − *I*_║_)/(*I*_⊥_ + *I*_║_), where *I*_⊥_ (*I*_║_) denotes the PL intensity measured with linear polarization perpendicular (parallel) to the NW axis.

## Results and Discussion

### Alloy disorder and band-tail states

**Figure 2:**
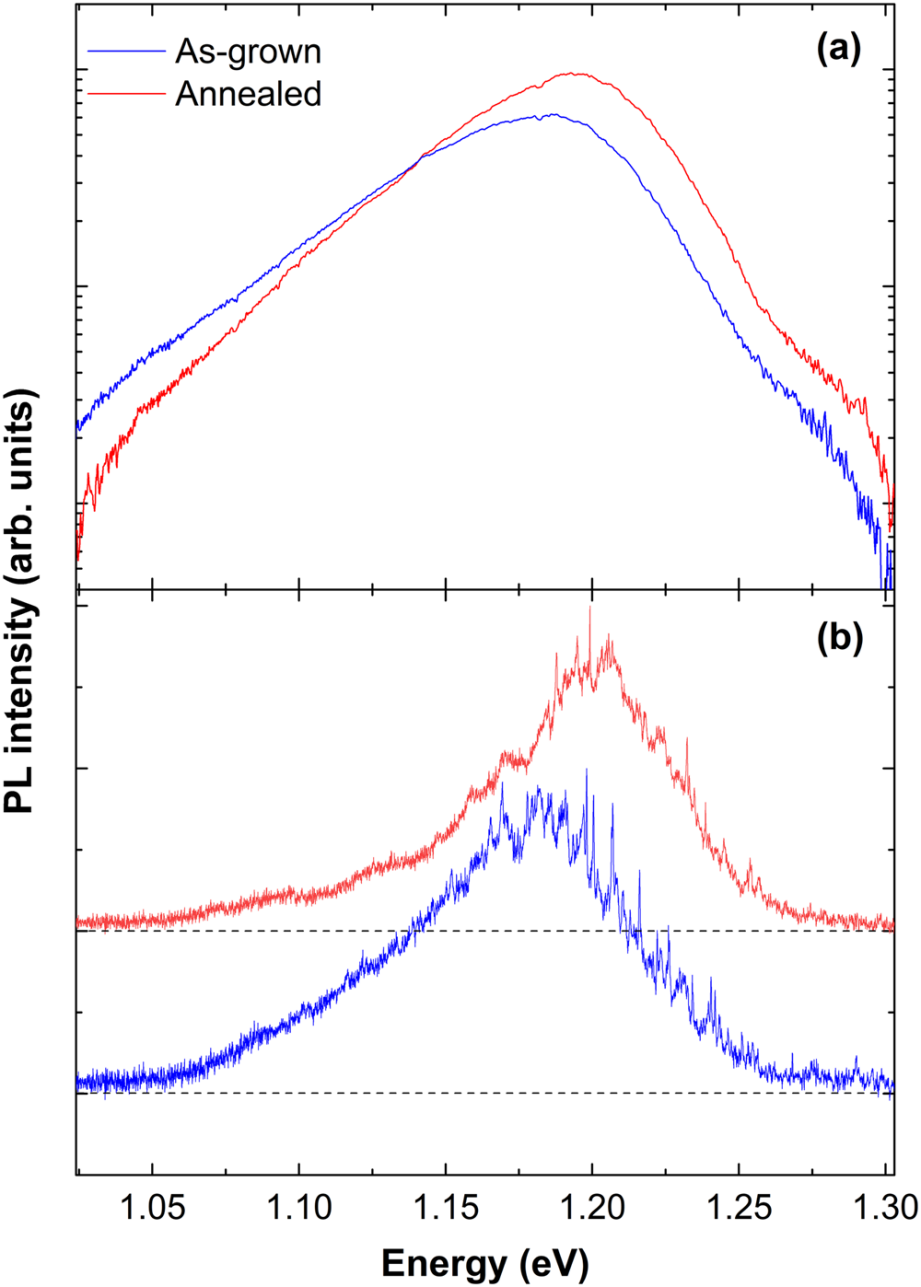
Low-temperature PL spectra. (**a**) PL spectra of as-grown (blue) and annealed (red) NW ensembles at 5 K plotted in semilogarithmic scale. (**b**) Representative PL spectra of single as-grown (blue) and annealed (red) NWs at 5 K. Single NW spectra are normalized to the same maximum intensity and are shifted vertically for clarity.

Highly-mismatched alloys, such as GaNAs studied in this work, are known to be prone to high alloy disorder, as even small compositional fluctuations result in large variations of the bandgap energy that are amplified due to the giant bandgap bowing. The compositional fluctuations can be evaluated by PL spectroscopy performed at low temperatures^52^, i.e. under conditions when the photoexcited carriers rapidly thermalize to the band-tail states. Low-temperature PL spectra from the as-grown and annealed NW arrays are presented in Fig. 2a. In both structures, the PL spectra are rather broad and have an exponential tail on the low energy side, which is common for localized exciton (LE) emissions in GaNAs structures^52,53^. The high-energy cut-off of the spectra corresponds to the bandgap energy of the GaNAs alloy with the intended N composition of 2%. Therefore, we can conclude that the optically active region of the studied CSS NWs is the GaNAs shell with a smaller bandgap. Post-growth annealing has several effects on the PL emission. First of all, it leads to an increase in the PL intensity, which was previously attributed to annealing-out of non-radiative defects^47^. Secondly, it causes a change in the low-energy slope of the PL peak. Since at low temperatures this slope reflects the localization potential in the GaNAs shell caused by alloy fluctuations^52,53^, the observed changes imply that the annealing decreases the localization energy, or, in other words, improves long-range uniformity in the alloy composition. This also explains the observed slight blueshift of the PL peak position. Similar effects have previously been reported in GaNAs epilayers^45,46^.

In addition to long-range alloy fluctuations, the GaNAs alloy is also known^40,54^ to exhibit short-range compositional fluctuations, where a local variation of nitrogen content is strong enough to result in three-dimensional (3D) confinement of excitons. To examine the effect of post-growth thermal annealing on these quantum dot (QD)-like regions, we have performed μPL measurements on single NWs transferred to another Si substrate. The representative results of these measurements are summarized in Fig. 2b. In both as-grown and annealed NWs, the μPL spectra contain numerous sharp lines superimposed on the broad LE background. The narrow linewidth of these lines suggests that they stem from the QD-like regions in GaNAs with the 3D exciton confinement. Based on the statistical analysis performed on several NWs, an average number of the QD emitters within individual NWs does not significantly change after annealing. This shows that annealing cannot suppress the short-range fluctuations in N content. It, however, affects the local strain within the QD regions as will be discussed below.

### Global strain

NW structures comprised of core and shell layers with different lattice constants are expected to experience strain^55–57^. This remains true for CSS structures such as the GaAs/GaNAs/GaAs NWs studied in this work. According to Vegard’s law, the lattice constant of GaNAs decreases with increasing nitrogen content. Therefore, the inner GaNAs shell should experience global strain. Following the formalism developed by Ferrand and Cibert^56^, the strain in the GaNAs inner shell contains two components: a uniform longitudinal component along the NW axis ${\varepsilon }_{zz}^{is}$ and an in-plane strain component ${\varepsilon }_{in-plane}^{is}=\,({\varepsilon }_{rr}^{is}+{\varepsilon }_{\theta \theta }^{is})/2$ that depends on the radial coordinate (*r*). They can be calculated using the following equations:

1 $${\varepsilon }_{zz}^{is}=(\eta -1)\cdot f,$$

2 $${\varepsilon }_{\theta \theta }^{is}(r)={B}_{is}\cdot {\left(\frac{{r}_{c}}{r}\right)}^{2}-(1-\eta )\cdot (f+{B}_{is}),$$

3 $${\varepsilon }_{rr}^{is}(r)=-\,{B}_{is}\cdot {\left(\frac{{r}_{c}}{r}\right)}^{2}-(1-\eta )\cdot (f+{B}_{is})$$

where ${r}_{c}$ is the average radius of the NW core. Here,

4 $$f=({a}_{is}-{a}_{c})/{a}_{c},$$

where *a*_c_ and *a*_is_ are lattice constants of pure GaAs core and GaN_0.02_As_0.98_ inner shell. The latter is calculated according to the Vegard’s law. The relative cross section of the inner shell $\eta$ is defined as:

5 $$\eta =({r}_{c+is}^{2}-{r}_{c}^{2})/{r}_{NW}^{2}$$

where ${r}_{c+is}$, ${r}_{NW}$ are the average radii of the NW core and inner shell and total radius of the NW, respectively. The parameter ${B}_{is}$ is derived under assumption of the equal stiffness constants (c_ij_) for GaAs and GaNAs:

6 $${B}_{is}=-\,f({c}_{11}+2{c}_{12})/({c}_{11}+{c}_{12}+2{c}_{44})$$

The calculations show that the longitudinal tensile strain ε_zz_ along the NW axis is expected to be quite high, around 0.31%, which dominates over the in-plane tensile component of 0.066%. The strain should cause splitting (Δ) between light-hole (lh) and heavy-hole (hh) valence subbands in the GaNAs shell, which can be estimated using the Bir-Pikus Hamiltonian, as described in Ferrand’s and Cibert’s work^56^. The calculated hh-lh splitting is found to be independent of the longitudinal coordinate and decreases radially from ∼30 meV at the core-inner shell boundary to ∼20 meV at the boundary between the inner shell and the outer shell.

**Figure 3:**
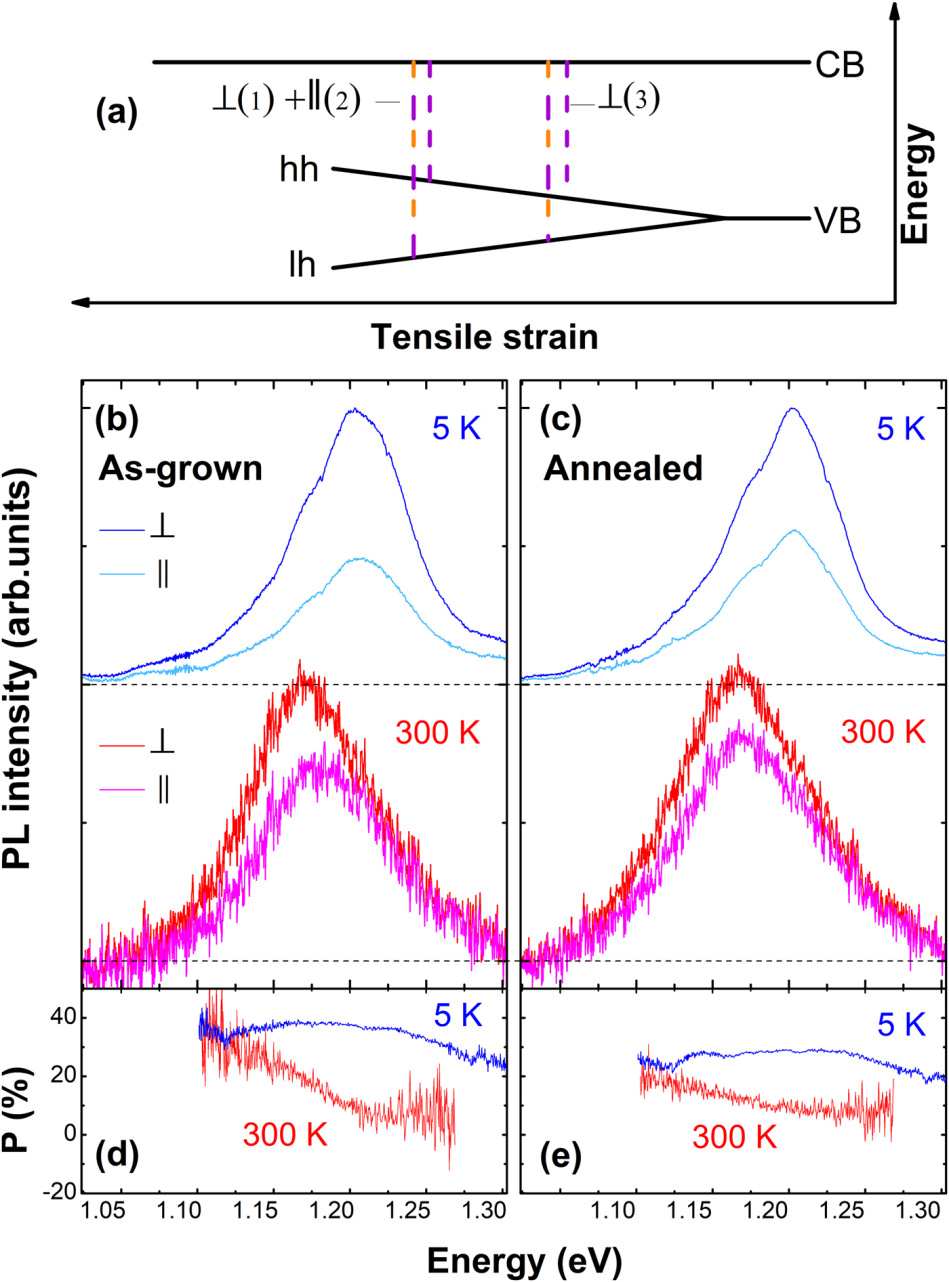
Strain-induced hh-lh splitting. (**a**) Schematic energy diagram of the valence band splitting with increasing tensile strain. The vertical dashed lines show the differently polarized band-to-band optical transitions involving the lh and hh states. Polarization-resolved PL spectra from the as-grown (**b**) and the annealed (**c**) NWs measured at 5 and 300 K, by detecting the PL components polarized orthogonally (the blue and red lines) and parallelly (light blue and magenta) to the NW axis. (**d** and **e**) show PL polarization degrees at 5 K (the blue line) and 300 K (the red line) from the as-grown and the annealed NWs, respectively.

The actual strain in the optically active GaNAs shell of the studied CSS NWs can be analyzed by studying polarization of the band-to-band optical transitions. This is because interband optical transitions between the CB and the valence subbands obey different selection rules, namely, the optical transitions involving the hh states are polarized orthogonally to the NW axis, while the lh-related transitions have their polarization directions both parallel and orthogonal to the NW axis. This is schematically illustrated in Fig. 3a, where ⊥ (║) denotes the optical transition with the orthogonal (parallel) polarization and the numbers given in parentheses indicate their relative oscillator strengths. Considering that the calculated hh-lh splitting is around 20–30 meV, we expect that only the hh states are populated at low measurement temperatures (e.g. 5K) in the as-grown structures, whereas the lh states can participate in emission at elevated temperatures (e.g. 300K).

This scenario is indeed confirmed from polarization-resolved μPL measurements performed on lying NWs and results of these measurements from the as-grown (annealed) structures are summarized in Fig. 3b,d (3c and 3e). The shown data were averaged over 30 individual as-grown and 30 annealed NWs that were transferred onto another Si substrate. From Fig. 3b,c it is obvious that at 5K the detected PL emission is preferably polarized orthogonally to the NW axis and that the maximum position is the same for the ⊥- and ║-polarized PL components. Moreover, no spectral dependence of the PL polarization degree P is observed - see Fig. 3d,e. This is consistent with the suggestion that only the hh-states participate in the emission at 5K due to the large hh-lh splitting. At room temperature (RT), however, a noticeable shift of the ║-polarized PL component is observed in the as-grown NWs, by ∼11 meV towards a higher energy as seen in Fig. 3b. This is accompanied by a decrease of the measured polarization degree P, which now exhibits a strong spectral dependence. Such behavior suggests that at RT the thermal energy is sufficient to facilitate redistribution of holes between the hh and lh subbands so that both of them take part in the radiative transitions. (We note that at 5K the measured P is lower than the expected value of 100%. This is tentatively attributed to PL depolarization due to, e.g., scattering of the emitted light at the rough NW surface and also due to different spatial offset of the emitters relative to the NW center and coupling of the emitted light to far field^58^). Annealing causes a decrease of the polarization degree and a reduction of the splitting between the ⊥- and ║-polarized PL components to around 6 meV – see Fig. 3c,e. This provides direct experimental evidence for a reduction of the tensile strain in the GaNAs shell of the annealed NWs.

We can model the RT polarization-resolved PL spectra by taking into account a thermal redistribution between the hh and lh states, using the GaNAs bandgap *E*_g_ calculated within the BAC model and taking spectral broadening from the measured data. For the as-grown samples, such simple estimate yields Δ = 17 meV, which is only slightly lower than the calculated value given above. This may be partly related to partial strain relaxation during the growth through e.g. the tilted stacking-faults observed in transmission electron microscopy studies, shown in the Supplementary Figs. S1 and S2, and to the unintentional formation within the GaAs core of an additional thin layer with a low N content due to the specifics of the growth process^49^. After the annealing Δ is reduced to Δ = 12 meV, reflecting partial strain relaxation, likely due to the formation of strain-relaxing defects. Indeed, scanning electron microscopy studies (shown in Supplementary Fig. S3) show the appearance of several pits and extended defects on the NW surface after the annealing treatment, likely causing a reduction of the global strain. In fact, similar extended defects have been reported to appear in GaNAs epilayers grown on GaAs after annealing^59^. We note that the related defects do not have detrimental effects on the radiative efficiency of the investigated NWs as the PL intensity is higher in the annealed structures.

### Local strain

Annealing can affect not only global but also local strain, i.e. the strain experienced by QD-like emitters at short-range fluctuations of the N composition. The local strain can be probed by studying polarization properties of the QD-like emission lines detected from single NWs – see Fig. 2b. This is because the electronic structure of excitons trapped within these QD states critically depends on the local strain: the hole confined in a QD emitter is of a pure hh character under high tensile strain directed along its principal axis. The hh QD emitters with the symmetry of C_3v_ or higher will emit a single line that stems from the degenerate |M > = | ± 1> exciton states and is polarized in the plane orthogonal to the high-symmetry QD axis. On the other hand, holes confined in QDs that experience low (or no) local strain will have mixed hh-lh character due to the reduced hh-lh splitting. Such QDs are expected to emit a pair of orthogonally polarized lines from the bright |J, M > = | 1,0> and |1,±1> exciton states with a rather large splitting.

**Figure 4:**
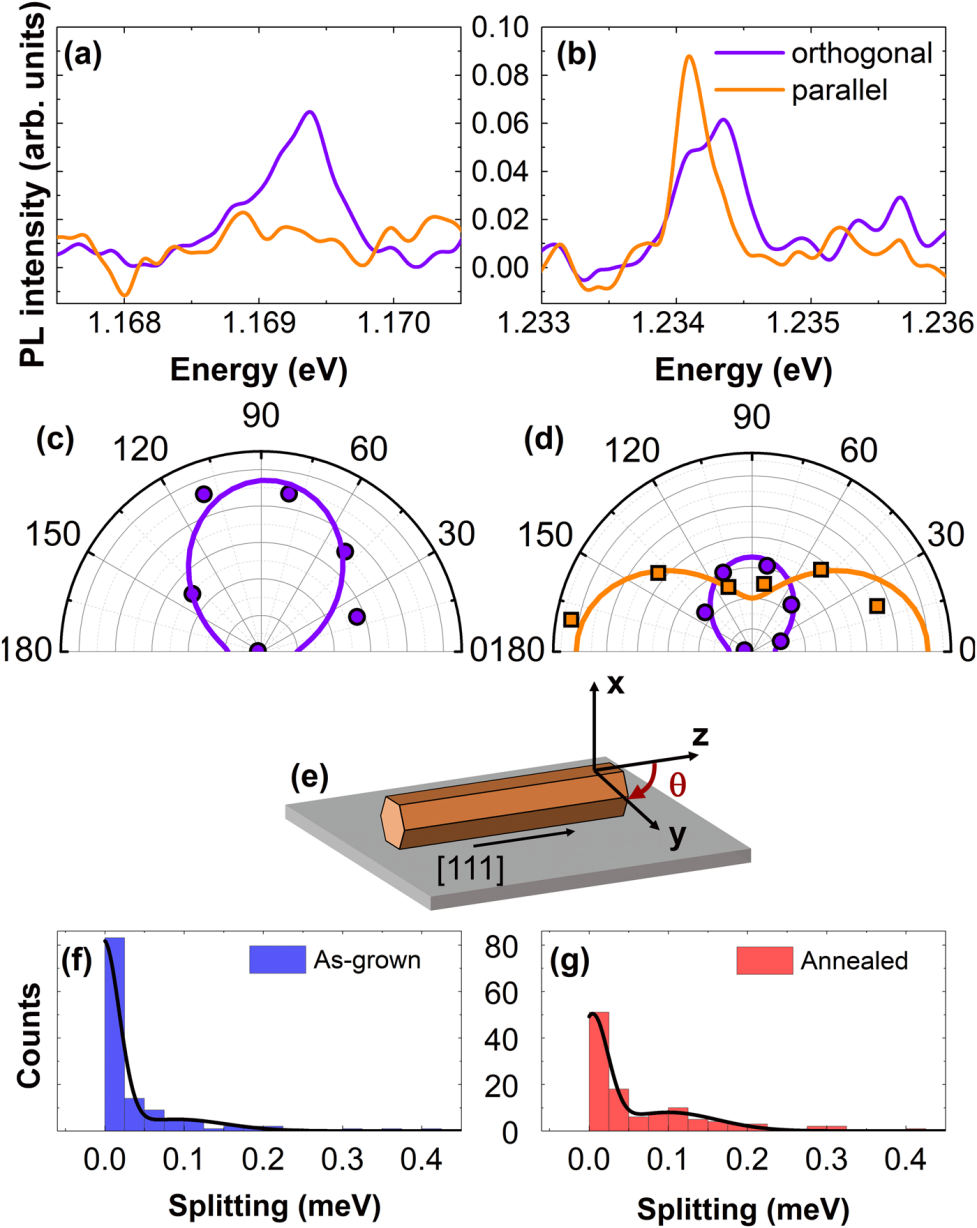
The effect of strain on the QDs. Representative polarization-resolved single QD-like emission lines with the polarization axis orthogonal to the NW axis *z* (**a**) and with a split pair of orthogonally polarized lines (**b**). (**c**) and (**d**) show the corresponding polar plots of line intensities for the lines shown in (a) and (b), respectively. The circles and squares show intensities of the PL line polarized along *y* and *z* axis, respectively, with the axes and polarization detection angle θ as defined in (**e**). (**f**) and (**g**) show distributions of the splitting between the two orthogonally polarized QD-like emission lines in the as-grown and the annealed samples, respectively, collected from 125 QD-like lines for each structure. The solid lines are the fitting curves of the distribution using the Gaussian function.

Figure 4a,b show examples of the polarization-resolved μPL spectra of the QD emitters. Two types of the QD emissions can be distinguished: (i) one single emission line polarized orthogonally to the NW axis, with P ranging between 40 and 100% (Fig. 4a), and (ii) a pair of orthogonally polarized lines (Fig. 4b) separated by more than 50 µeV. The corresponding polar plots, showing the polarization-resolved μPL intensity as a function of angle θ, are shown in Fig. 4c,d, respectively. θ represents the angle of the linear polarizer axis relative to the NW axis, where θ = 0 corresponds to detection of light polarized parallel to the NW axis (see Fig. 4e). Since the principal axes of the QDs formed in GaNAs NWs are preferentially aligned along to the NW axis^40^, these characteristics suggest that the sharp lines of type (i) originate from the QDs with the pure hh character whereas the type (ii) lines stem from the QD emitters with the mixed hh-lh character.

Annealing does not reduce the number of the formed QDs. However, it changes their electronic structure. This can be seen by comparing Fig. 4f,g, which show statistical distributions of QDs with different energy splitting between the orthogonally-polarized PL components for the as-grown and annealed NWs, respectively. The QDs in the as-grown NWs generally exhibit a small energy splitting (or only emit one strongly polarized PL line), as expected for the hh-QDs of high symmetry. On the other hand, a significant number of the QDs in the annealed NWs emit a pair of lines separated by more than 50 µeV and, hence, have the mixed hh-lh character. The formation of type (ii) QDs in the annealed NWs is thus attributed to a reduction of local strain, and thus the hh-lh splitting in the QDs. Therefore, we suggest that besides the aforementioned change in the global core-shell strain, annealing also reduces the local strain within the short-range fluctuation in N composition, likely due to combined effects of reduced global strain and improved uniformity in the N distribution within the GaNAs shell.

## Summary

In summary, we have employed polarization-resolved μPL spectroscopy to investigate effects of post-growth annealing on alloy fluctuations and strain variations in the MBE-grown GaAs/GaNAs/GaAs CSS NWs. It is found that annealing improves alloy uniformity by suppressing the long-range fluctuations in the N composition, evident from the reduction of the localization energy that characterizes the band-tail states. It also reduces the local strain within the QD-like regions caused by short range fluctuations in the N composition, judging from the observed changes in the electronic structure of the related QD emitters. In addition, partial relaxation of the global strain caused by the lattice mismatch between the GaNAs and GaAs is observed and is tentatively attributed to the formation of structural defects. These defects, however, do not affect optical quality of the alloy, as the radiative efficiency of the studied NWs is enhanced in the annealed structure. The obtained results are of importance for improving optical quality and device applicability of NWs from highly-mismatched alloys. Moreover, the revealed relaxation of the global strain due to post-growth annealing should be taken into account when utilizing strain engineering in NW heterostructures, as such annealing often occurs during device fabrication and processing steps.

## Supplementary information

Supplementary information.

## Data Availability

The datasets generated during and/or analyzed during the current study are available from the corresponding author on reasonable request.
