# Supplementary material for: Effects of thermal annealing on localization and strain in core/multishell GaAs/GaNAs/GaAs nanowires
Source: Sci Rep. 2020 May 19;10:8216. doi: 10.1038/s41598-020-64958-6 (PMC7237432; doi:10.1038/s41598-020-64958-6)
Supplement: Supplementary file 1 — Supplementary information. [file 41598_2020_64958_MOESM1_ESM.pdf]

Supplementary information

Effects of thermal annealing on localization and strain in core/multishell GaAs/GaNAs/GaAs nanowires

Roman M. Balagula[1,‡], Mattias Jansson[1,‡,*], Mitsuki Yukimune[2], Jan E. Stehr[1], Fumitaro Ishikawa[2], Weimin M. Chen[1], & Irina A. Buyanova[1]

[1] *Department of Physics, Chemistry and Biology, Linköping University, 581 83 Linköping, Sweden*

[2] *Graduate School of Science and Engineering, Ehime University, 790-8577, Matsuyama, Japan*

[‡] These authors contributed equally to this work

1. Transmission electron microscopy

To investigate the structure of the NWs, transmission electron microscopy (TEM) was performed on individual NWs transferred to a TEM-grid, using a FEI Tecnai G2 TF 20 UT transmission electron microscope.

Figure S1a shows a representative overview TEM micrograph of an annealed GaAs/GaNAs/GaAs NW. Numerous lines normal to the NW growth axis are observed, corresponding to stacking faults (SF) and rotational twin planes, which are very commonly observed in III-V NWs. Moreover, several additional structural defects in the form of SFs tilted away from the NW growth axis by approximately 20°, were also observed, as exemplified by the magnifications. As can be seen in Fig. S1b and c, where TEM micrographs of NWs before annealing are shown, the same type of defect is present also in NWs from this sample. A HRTEM micrograph of one such defect is shown in Fig. S2, where the SF is indicated with the red dot.

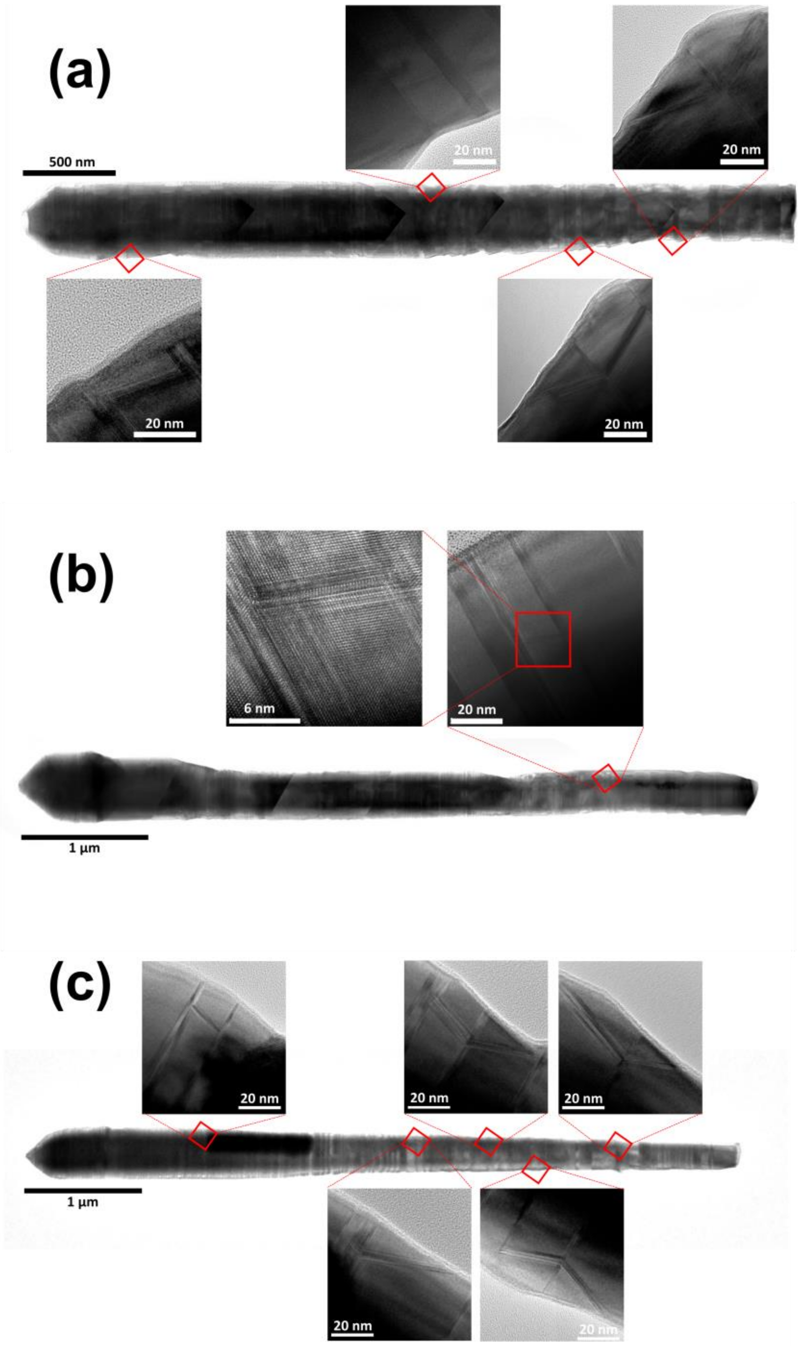

Supplementary figure S1: TEM micrographs of an annealed NW (a) and NWs before annealing (b),(c). The magnifications show the tilted SFs.

From this preliminary investigation, no definitive conclusion regarding the role of these structural defects in the annealing-induced strain reduction could be drawn, as it is not clear whether the number or extent of the defects change with annealing. In fact, the number of tilted SFs varies significantly between NWs, as seen in Fig. S1b and c, where one and five defects were found in the two respective NWs.

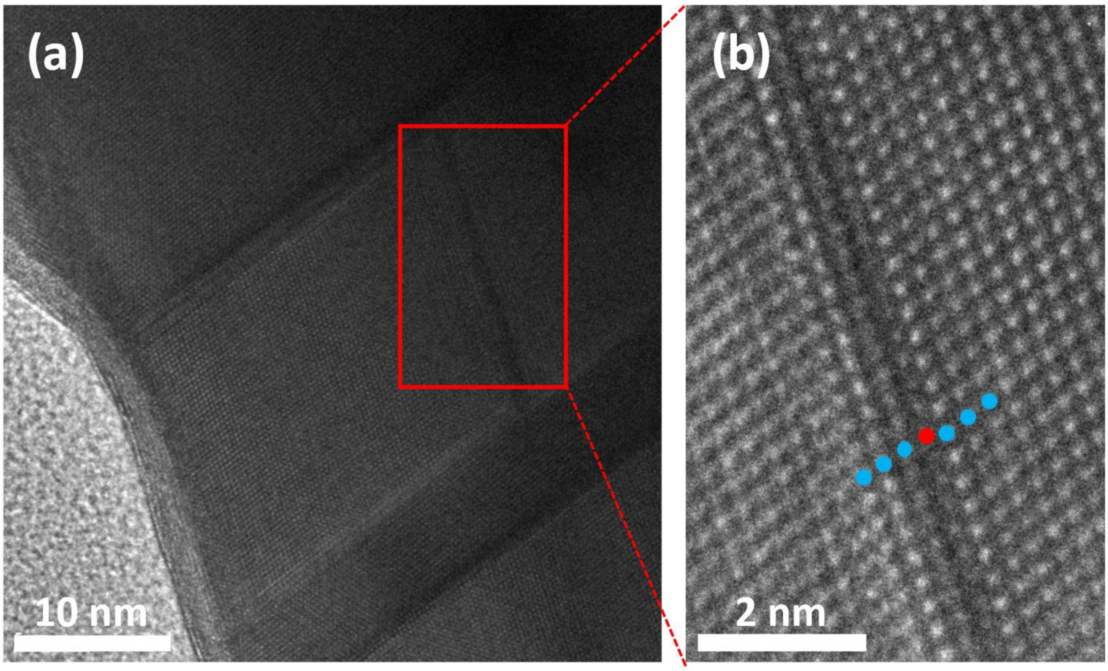

Supplementary figure S2: A TEM image of a representative tilted SF (a), and a high-resolution TEM image of the same defect, emphasized with the blue and red dots.

2. Scanning electron microscopy

Figure S3a and b shows scanning electron microscopy (SEM) images acquired from NWs before (a) and after (b) annealing, using a LEO 1550 Gemini scanning electron microscope. In Fig. 3b, a number of pits and extended longitudinal defects appear on the surface of the NWs, which evidently form upon annealing. Figure 3c shows a magnification of a representative annealed NW, where the defects can be clearly seen, and the extended longitudinal defects are indicated by the white arrows. Similar,

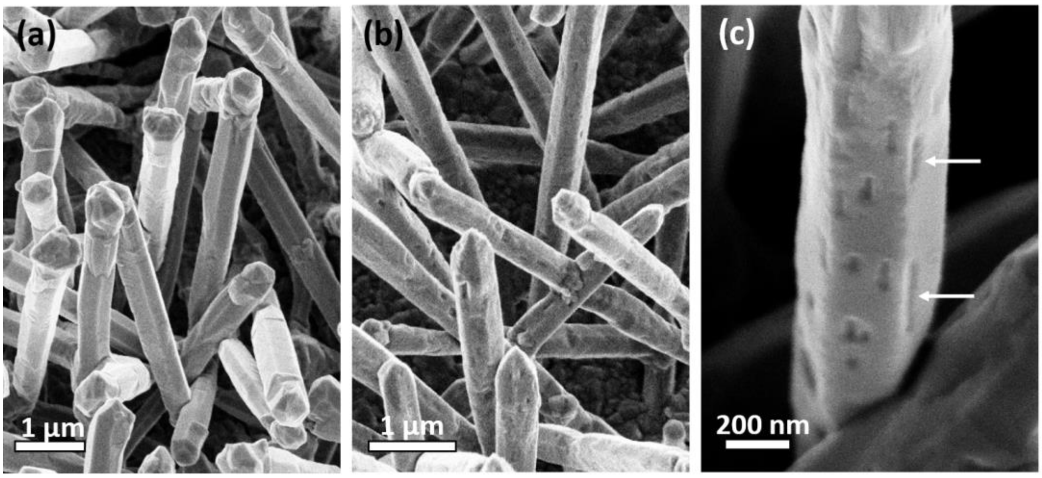

Supplementary figure S3: SEM micrographs of NWs from the samples without (a) and after (b) the annealing. (c) shows a magnification of an annealed NW, where the pits are clearly seen. Some pits evolve into vertical lines, as indicated by the arrows.

extended defects have been shown to appear in similarly highly strained GaNAs epitaxial layers grown on GaAs, upon annealing treatment[See ref. 59 of the main text]. While the exact nature of the defects is not yet known, we speculate that they may be tell-tale signs of strain-reducing dislocations.
